# Improved detection of isoniazid-heteroresistant *Mycobacterium tuberculosis* subpopulations by droplet digital PCR compared to MeltPro TB assay

**DOI:** 10.1128/spectrum.00030-25

**Published:** 2025-08-26

**Authors:** Haoran Li, Nan Wang, Mengjie Yang, Yuanyuan Shang, Yilin Wang, Chianru Tan, Shufang Wen, Yong Guo, Yu Pang

**Affiliations:** 1 Department of Bacteriology and Immunology, Beijing Tuberculosis and Thoracic Tumor Research Institute/Beijing Chest Hospital, Capital Medical University 12517, Beijing, People’s Republic of China; 2 School of Biomedical Engineering, Tsinghua University 12442, Beijing, People’s Republic of China; Griffith University-Gold Coast Campus Institute for Glycomics, Gold Coast, Australia

**Keywords:** *Mycobacterium tuberculosis*, isoniazid, droplet digital PCR, heteroresistance

## Abstract

Drug resistance in *Mycobacterium tuberculosis* (Mtb), especially isoniazid (INH) resistance, challenges tuberculosis control. This study evaluated droplet digital PCR (ddPCR) against the traditional MeltPro TB assay. A total of 77 INH-resistant samples from Beijing Chest Hospital, China, underwent ddPCR, drug susceptibility testing, and Sanger sequencing. Of the 73 valid samples, MeltPro detected 55 INH-resistant and 18 heteroresistant samples; ddPCR found 55 high-frequency mutations, 11 of the 18 heteroresistant by MeltPro as heteroresistant, and 6 of the 7 below limit as sensitive, 1 as *Nocardia*. Using ddPCR as a secondary screening tool for MeltPro results can screen false positives in MeltPro, improving INH resistance detection accuracy (98.63% vs. 89.04%). DdPCR technology performs excellently in tuberculosis drug resistance detection and provides strong technical support for the accurate diagnosis and treatment of tuberculosis.

**IMPORTANCE:**

Tuberculosis has emerged as a significant threat to global health, bringing numerous new cases and a large number of deaths annually. Isoniazid (INH), as a first-line treatment drug, plays a crucial role. However, the emergence of its drug resistance poses a tough challenge for tuberculosis control. Currently, the methods for detecting INH resistance and heteroresistance have limitations like the slow speed of traditional culture tests and the insufficient sensitivity of molecular methods. Therefore, improving the diagnostic efficacy of the detection results for INH resistance is crucial for the treatment of tuberculosis.

## INTRODUCTION

Tuberculosis (TB), as the leading cause of death from a single infectious agent in the world, poses a serious threat to global health (1). According to the WHO statistics, the number of new TB cases worldwide reached 10.8 million in 2023, among which China reached 0.74 million, ranking third in the world (2). Although there are currently many anti-TB drugs that can cure the disease relatively well, due to the increased drug pressure, increasingly *Mycobacterium tuberculosis* (Mtb) increasingly evolves selectively during the treatment process (3, 4). A few bacterial colonies first undergo drug-resistant mutations, putting the Mtb colonies in the body in a heteroresistance state and eventually breaking out completely without being detected, eventually developing into drug-resistant TB (5). Among them, isoniazid (INH), as the core component of the first-line anti-TB drug regimen, the increasing drug resistance to this drug undoubtedly brings challenges to the treatment and diagnosis of TB (6).

The classical proportion method, a primary way to determine the heteroresistance of TB, mainly depends on the ratio of the colony count on the drug-containing medium to that on the drug-free one (5). The threshold for distinguishing sensitive from resistant strains of INH is 1% (7). Methods must identify at least 1% resistant bacteria to avoid misclassifying resistant samples as sensitive, which could lead to improper medication use and drug-resistant TB development. Traditional culture tests are practical but slow, while molecular methods can shorten detection time but often miss low-abundance heteroresistance (8). For instance, DeepMelt and Sanger sequencing need at least 15–20% mutants, and MeltPro TB/INH, a clinically approved method, detects only 20–40% of INH heteroresistance (9–12). Although MeltPro is currently widely used in clinical detection, numerous studies have shown that MeltPro has a relatively high false positive rate, and approximately 10% of true negative samples are misdiagnosed as positive (13, 14). Hence, there is an urgent need for the emergence of novel molecular biology technologies to optimize further or even surpass the existing drug resistance detection techniques.

Droplet digital PCR (ddPCR) technology has higher sensitivity, specificity, and quantitative accuracy than traditional PCR technology (15, 16), and has been generally recognized for sensitive detection of rare mutations, especially in the presence of high wild-type gene abundance, such as tumor liquid biopsy and prenatal diagnosis (17, 18). Previous studies showed that ddPCR technology is more sensitive than commercialized MeltPro TB/INH in detecting INH resistance and can detect samples with INH heteroresistance. However, its single detection only “saved” two missed diagnoses (MeltPro TB/INH vs ddPCR, 8/49 vs 6/49). In another cohort, ddPCR had a higher positive detection rate due to better heteroresistance detection (MeltPro TB/INH vs ddPCR, 9/21 vs 14/21) (19). On the premise that MeltPro, using ddPCR as a secondary screening tool, will have the potential to conduct further precise screening for drug resistance and heteroresistance, thus avoiding the occurrence of misdiagnosis. Therefore, we included 77 TB patients who showed INH resistance in MeltPro TB testing and further performed ddPCR to confirm INH resistance. And the optimization potential of the ddPCR reexamination of both in the diagnosis of INH resistance was explored.

## MATERIALS AND METHODS

### DNA template and sample collection

Plasmids were designed and synthesized with reference to the NCBI database and contained the amplicon sequence of the target gene (Sangon, Shanghai, China), the *katG* (NC_000962.3, 2154916-2155315) plasmids included wild-type S315 (AGC) and common mutant types, S315T (AGC→ACC), S315R (AGC→AGA/AGG/CGC), and S315I (AGC→ATC), and the *inhA* promoter (NC_000962.3, 1673299-1673497) plasmids include wild-type and common mutant types (−15C→T, −8T→C).

This retrospective study enrolled 77 patients diagnosed with INH-resistant TB by the MeltPro TB test in Beijing Chest Hospital in China from August 2022 to April 2023. Both sputum specimens and their corresponding Mtb cultures are taken from the biobank of Beijing Chest Hospital. A waiver of informed consent was obtained given the study’s retrospective nature.

### DNA extraction

DNA of Mtb was extracted from 1 mL decontaminated specimens (sputum and their corresponding mycobacterial cultures) using the Sanity 2.0 TB DNA Extraction Kit (Zeesan Biotech, Xiamen, China) on an automated DNA extraction system (Zeesan Biotech, Xiamen, China), following the manufacturer’s instruction. The same DNA extract was subsequently analyzed by both MeltPro assay and ddPCR.

### MeltPro TB

5 µL of the crude DNA was introduced into the PCR mixture containing self-quenching fluorescent probes labeled with 6-FAM and 6-TET. The LightCycler 480 system (Roche Applied Science, IN, USA) analyzed PCR amplification and melting curve. The following protocol carried out the polymerase chain reaction: at 50°C, uracil-N-glycosylase was used for decontamination treatment for 2 min; denaturation was performed at 95°C for 5 min; a touchdown program of 10 cycles was executed, including maintaining at 95°C for 10 s, 25 s at 71°C (reducing 1°C per cycle), and maintaining at 75°C for 30 s, followed by 45 cycles, encompassing maintaining at 95°C for 10 s, 25 s at 61°C, and 25 s at 75°C. The melting curve analysis was initiated by a 2-min denaturation step at 95°C, followed by hybridization at 40°C for 2 min. The temperature was gradually raised from 40°C at a rate of 1°C per step to 85°C, with a 5-s pause between each step.

### ddPCR assay

DdPCR was performed via Mtb INH resistance mutation detection kit (TargetingOne, Beijing, China) and the ddPCR System D2 (TargetingOne, Beijing, China) which consists of DropMaker M1 and ChipReader R2, the detection kit uses the drop-off probe design for INH resistance testing (20). The 30 µL PCR reaction mixture included 7.5 µL 4 × Taq DNA polymerase Supermix, 3 µL primers and probes mix (final concentrations 600 and 300 nM respectively), 2 µL internal control standard, 2.5 µL nuclease-free water, and 15 µL DNA template. Droplet generation chip contains the PCR mixture and 180 µL droplet generation oil in DropMaker M1 during the droplet generation procedure. The generated droplets were then collected by an 8-strip PCR tube before amplification. The thermal cycler with conditions as follows: pre-denaturation at 95°C for 10 min; amplification for 45 cycles with denaturation at 94°C for 30 s and annealing at 56°C for 1 min. After the amplification, the detection chip was combined with the PCR tube and placed in the ChipReader R2 to perform the detection procedure. The detection results were analyzed by ChipReaderR2 (V1.0.0.0) software, and all processes were performed according to the kit instructions. For the drop-off design, the droplet cluster could be classified as wild-type or mutant based on the fluorescence amplitude of the two fluorescent probes. The mutation rate was then calculated according to the formula:


$$katG\;S315\;mutation\;rate=\frac{\text{VIC copies}-\text{FAM copies}}{\text{VIC copies}}$$

$$inhA\;promoter\;mutation\;rate=\frac{\text{Cy5 copies}-\text{ROX copies}}{\text{Cy5 copies}}$$

wherein VIC copies represent *katG* reference gene copy number or the total number of genomes, FAM copies represent the number of genomes excluding the *katG* mutation site. Similarly, Cy5 copies represent *inhA* promoter reference gene copy number or the total number of genomes, ROX copies represent the number of genomes excluding the *inhA* promoter mutation site.

### Analytical evaluation of the ddPCR

To evaluate the performance of ddPCR for detecting different katG and inhA mutant types, we tested mutant plasmids (S315N-AAC, S315T1-ACC, S315R1-AGA, S315R2-AGG, S315R3-CGC, S315I-ATC, *inhA* 15C-T, and *inhA* 8T-C) alongside H37Rv genomic DNA. Mock samples were prepared at varying heterogeneity rates (1%, 5%, and 50%).

The limit of blank (LOB) was calculated separately for different genomic DNA concentrations (100, 1,000, and 10,000 copies per reaction) using 20 replicates for each concentration. The LOB was defined as the mean false-positive percentage plus its 95% confidence interval (CI).

To determine the limit of detection (LOD), we prepared reactions containing 10,000 genomic DNA copies, consisting of either the *katG* S315T (AGC→ACC) or *inhA* promoter 15C-T mutant plasmid mixed with the H37Rv genome at heterogeneity rates of 0.16%, 0.32%, 0.63%, 1.25%, 2.5%, 5%, 10%, and 20%. Each heterogeneity level was tested in triplicate. The LOD was defined as the lowest heterogeneity rate that could be significantly distinguished from the LOB, as confirmed by 20 replicate measurements.

### DNA sequencing

Two fragments, *katG* and *inhA* promoter, were amplified through PCR, and the retrieved amplicons were submitted for Sanger sequencing. Regarding mutant-enriched Sanger sequencing, the DeepMelt protocol was adopted for PCR amplification to generate the amplicons for Sanger sequencing. Briefly, during the PCR amplification of both wild-type and mutant templates, a clamping probe was employed to impede the chain elongation of the wild-type template specifically, but it did not hamper the chain elongation of the mutant template. Consequently, the mutant template was enriched after PCR.

### Drug susceptibility test

Samples were subjected to decontaminating treatment with the N-acetyl-l-cysteine/NaOH method for 15 min, followed by a neutralizing process with sterile phosphate-buffered saline (PBS, pH 6.8) and a 15-min centrifugation at 3,000 × *g*. The resulting pellet was resuspended in 2 mL of PBS buffer. A 0.5 mL fraction of the decontaminated samples was then subjected to liquid culture using the MGIT 960 system. The drug susceptibility of the culture-positive isolates was ascertained in line with the standard proportion protocol on L-J solid medium. INH heteroresistance was defined as more than 1% colonies growing on the L-J medium containing 0.2 mg/mL of INH in contrast to the growth on the drug-free control medium.

### Statistical analysis

ddPCR performance evaluation and data analysis were performed using GraphPad Prism 8.0 (GraphPad Software, USA), and LOB determination for ddPCR was analyzed using IBM SPSS Statistics 23 (IBM, USA).

## RESULTS

### Study population

The study population included 73 participants with INH-resistant TB. As shown in Table 1, the median age was 51.6 years (ranging from 15 to 87), with 27 (36.99%) in the 15–44 age group, 20 (27.40%) in 45–60, and 26 (35.62%) over 60. Males accounted for 58 (79.45%) and females 15 (20.55%). Acid-fast staining results showed 6 (8.22%) negative (0/50 fields), 5 (6.85%) ± (1–3/50 fields), 20 (27.40%) 1+ (11–99/50 fields), 12 (16.44%) 2+ (1–9/1 field), 21 (28.77%) 3+ (10–99/1 field), and 9 (12.33%) 4+ (>100/1 field). By MeltPro detection, 55 (75.34%) were INH-resistant, and 18 (24.66%) showed INH heteroresistance. Additionally, 32 (43.84%) had a prior TB history, 27 (36.99%) had diabetes, 22 (30.14%) had liver disease, 15 (20.55%) had kidney disease, and 9 (12.33%) had hypertension/CHD. Notably, 40 (54.79%) had previous INH treatment, while HIV co-infection was undetected in 57 (78.08%) and unknown in 16 (21.92%). Negative conversion status was known in 27 (30.14% yes, 6.85% no) and unknown in 46 (63.01%).

**TABLE 1:** Demographic characteristics of participants^*a*^

Parameters	INH resistant (*n* = 73) (100%)
Age, years, median	51.6 (15–87)
15–44	27 (36.99)
45–60	20 (27.40)
>60	26 (35.62)
Sex	
Male	58 (79.45)
Female	15 (20.55)
Acid-fast staining	
- (0/50 visual fields)	6 (8.22)
± (1–3/50 visual fields)	5 (6.85)
1+ (11–99/50 visual fields)	20 (27.40)
2+ (1–9/1 visual fields)	12 (16.44)
3+ (10–99/1 visual fields)	21 (28.77)
4+ (>100/1 visual fields)	9 (12.33)
Tuberculosis history	
Yes	32 (43.84)
No	41 (56.16)
HIV co-infection	
Yes	0 (0.00)
No	57 (78.08)
Undetected	16 (21.92)
Complication	
Diabetes mellitus	27 (36.99)
Hypertension/CHD	9 (12.33)
Liver disease	22 (30.14)
Kidney disease	15 (20.55)
History of INH treatment	
Yes	40 (54.79)
No	33 (45.21)
Negative conversion	
Yes	22 (30.14)
No	5 (6.85)
Unknown	46 (63.01)
Detected by MeltPro	
INH resistance	55 (75.34)
INH heteroresistance	18 (24.66)


^*a*^ INH, Isoniazid.

### Analytical evaluation of ddPCR for INH resistance

**Fig 1:**
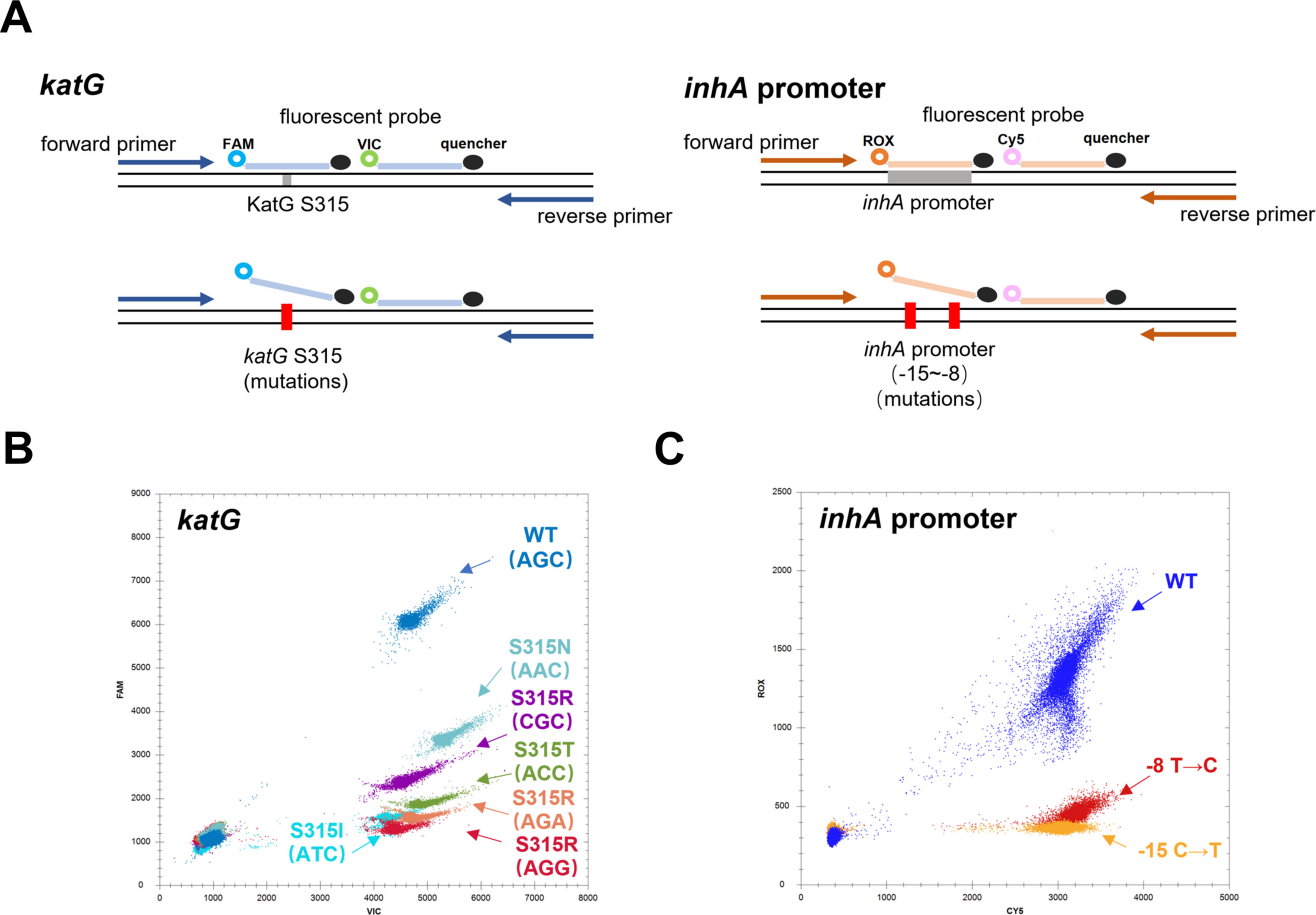
Schematic diagram of the principle and results of droplet digital PCR isoniazid resistance detection. (**A**) The detection of *katG* 315 was performed by FAM and VIC fluorescent probes, and the detection of position −15 to −8 of *inhA* promoter region was performed by ROX and Cy5 fluorescent probes. (**B**) Detection of different types of *katG* 315 mutation and wild type. (**C**) Detection of different types of *inhA* promoter mutation and wild type.

DdPCR performs a 5-plex assay in one PCR tube, the *katG*315, *katG* reference sequence, *inhA* promoter −15 to −8 region, *inhA* promoter reference sequence, and internal standard. Wild-type and mutant were distinguished by using a drop-off design. FAM and VIC fluorescent probes were used for the detection of the *katG* gene (Fig. 1A). When detecting the wild template, both FAM and VIC fluorescent probes can bind to the template and produce a double positive FAM/VIC signal. The mutant template will result in the drop-off of the FAM fluorescent probe, and the FAM fluorescent signal would be reduced or absent, allowing distinguishing from the wild type. The *inhA* gene was similarly detected by ROX and Cy5 fluorescent probe (Fig. 1B), the identifiable mutant types in this study included *katG* 315 mutant types: AGC-AAC, AGC-CGC, AGC-ACC, AGC-AGA, AGC-AGG, AGC-ATC, *inhA* promoter mutant type: -15C-T, -8T-C, which mutations cover almost all common drug-resistant mutation types (21).

**Fig 2:**
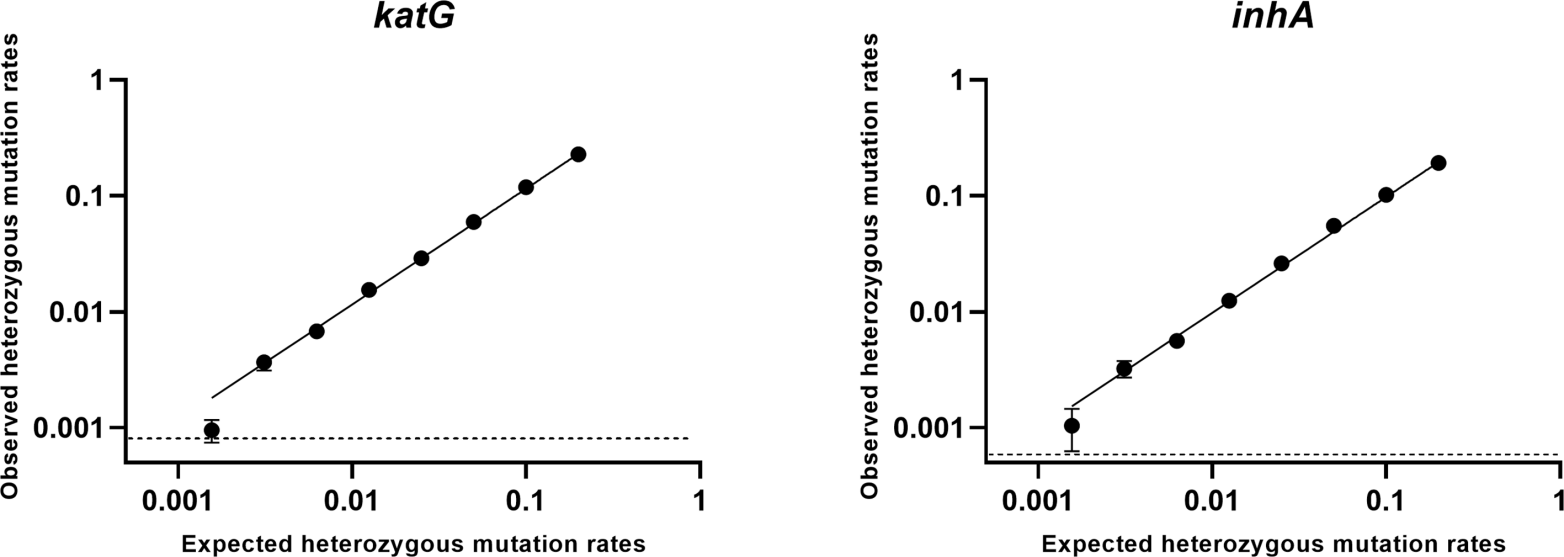
Limit of detection (LOD) results of katG (*katG* S315T AGC-ACC) and *inhA* promoter (-15C-T) at different percentages of heterozygous mutation rates (0.16%, 0.32%, 0.63%, 1.25%, 2.5%, 5%, 10%, and 20%), Dashed line, limit of blank (LOB).

DdPCR reliably quantified all *katG* and *inhA* mutant types at 1% heterozygosity (Fig. S1). The LOB analysis revealed concentration-dependent thresholds, with values of 0.081% for *katG* and 0.058% for *inhA* at 10,000 copies/reaction, 0.099% and 0.106% respectively at 1,000 copies/reaction, and 0.331% and 0.478% at 100 copies/reaction. The empirically determined LOD was 0.32% for *katG* S315T and 0.16% for the *inhA* promoter mutant (Fig. 2). Validation with 20 replicates confirmed all LOD results exceeded the LOB’s 95% CI threshold (Fig. S2).

### Diagnosis of INH resistance

**Fig 3:**
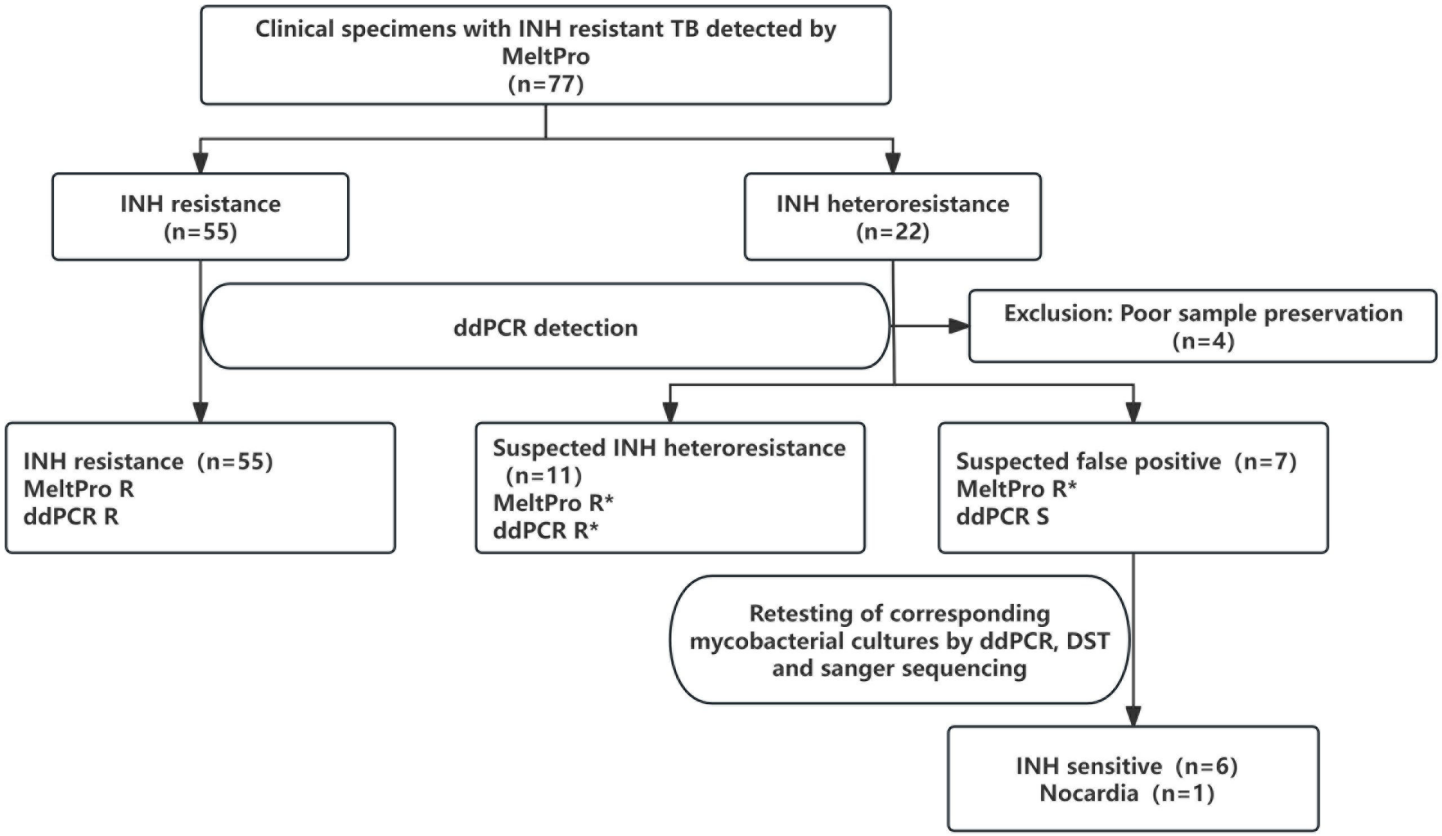
The workflow of this study. ddPCR, droplet digital PCR; INH, isoniazid; R, resistance; R*, heteroresistance; TB, tuberculosis.

As shown in Fig. 3, among the 77 clinical samples included, the MeltPro detection results indicated that 55 were resistant to INH, and 22 were heteroresistant to INH. Subsequently, using 100 copies of LOB as a reference, ddPCR-INH resistance detection was performed on these samples. Four samples were excluded as the bacterial quantity was extremely low, the internal reference gene was undetectable, and the drug resistance status could not be reported. Among the remaining 73 samples, 55 showed high-frequency INH resistance site mutations, consistent with the MeltPro diagnosis results. Among the 18 samples defined as heteroresistance by MeltPro, 11 were detected as heteroresistance and 7 were detected as sensitive by ddPCR.

To further verify these findings, we analyzed the seven discrepant samples using a combination of methods. For the six samples classified as INH-sensitive by ddPCR, drug susceptibility testing (DST) showed no growth of resistant colonies on INH-containing Löwenstein-Jensen medium, and Sanger sequencing of the *katG* and *inhA* regions revealed no resistance-associated mutations. These results are fully concordant with the ddPCR findings. The remaining sample was identified as *Nocardia* species by 16S rRNA sequencing, confirming all seven samples as false positives by MeltPro. Detailed clinical and laboratory characteristics of these samples are presented in Table 2.

**TABLE 2:** Information of false-positive samples^*a*^

Sample number	Age, years	Sex	Acid-fast staining	ddPCR (sputum)		ddPCR (mycobacterial culture)		Final diagnosis (ddPCR, DST, and sanger sequencing)
				Heterogeneity rate (*katG*)	Heterogeneity rate (*inhA*)	Heterogeneity rate (*katG*)	Heterogeneity rate (*inhA*)	
1	61	Male	1+	/	/	/	/	INH sensitive
2	63	Female	1+	/	/	/	/	INH sensitive
3	43	Male	±	/	/	/	/	INH sensitive
4	48	Female	±	/	/	/	/	INH sensitive
5	30	Male	±	/	/	/	/	INH sensitive
6	29	Male	0	/	/	/	/	INH sensitive
7	65	Male	0	/	/	/	/	Nocardia

^*a*^ ddPCR, droplet digital PCR; INH, isoniazid; /, undetected.

We subsequently established a composite reference standard incorporating ddPCR, DST, and Sanger sequencing results. Using this gold standard, we compared two diagnostic strategies: (i) MeltPro testing alone versus (ii) MeltPro testing with ddPCR confirmation. As shown in Table 3, the sequential testing approach significantly improved diagnostic accuracy for INH-resistant tuberculosis (98.63% vs 89.04% for MeltPro alone).

**TABLE 3:** Clinical performance of the ddPCR reexamination of INH-resistant samples^*a*^

Pilot	Final comprehensive diagnosis		Total	Accuracy (%) (95% CI)
	R	S		
Melt Pro				
MT	55	0	55	89.04 (0.80–0.96)
HET	10	8	18	
WT	0	0	0	
Total	65	8	73	
ddPCR reexamination				
MT	55	0	55	98.63 (0.95–1.00)
HET	10	1	11	
WT	0	7	7	
Total	65	8	73	

^*a*^ CI, Confidence Interval; ddPCR, droplet digital PCR; HET, heteroresistance; INH, isoniazid; MT, mutant type; R, resistance; S, sensitive; WT, wild type.

## DISCUSSION

The accurate identification of heteroresistance remains a persistent challenge in tuberculosis diagnostics (22). Traditional phenotypic DST typically requires 4–8 weeks for results and has limited sensitivity in detecting low-abundance (<10%) resistant subpopulations (23). Although molecular assays like MeltPro have substantially reduced turnaround times, they rely on melt curve analysis with a detection threshold of 30–50% mutation abundance, making them vulnerable to missed detection of low-level mutations and background noise interference (24). Available evidence indicates that low-level heteroresistance below 1% is clinically significant and strongly associated with treatment failure. Even a slight increase in the minimal inhibitory concentration (without reaching the critical concentration) can increase the risk of treatment failure by 84.3-fold (OR = 84.3, *P* < 0.001). Mechanistic studies show that low-frequency resistant subpopulations (<1%) rapidly expand under drug pressure. Mtb with Rv0678 mutations (initial frequency <1%) can develop full resistance within 6 months (25). In this context, developing more sensitive and specific diagnostic strategies is crucial for the precise management of drug-resistant tuberculosis. Initiating standardized treatment regimens containing RIF as early as possible for INH-resistant patients can reduce the risk of MDR-TB by 70–80%.^26^

DdPCR overcomes these limitations through its unique capabilities in droplet partitioning and absolute quantification (19). With the ability to detect mutation allele frequencies as low as 0.1%, it outperforms MeltPro by an order of magnitude, making it particularly effective in analyzing smear-negative samples from intermittently sputum-producing patients. ddPCR minimizes cross-reactivity with non-*Tuberculous mycobacteria*, enhancing diagnostic specificity. Its capacity for direct, standard-curve-free quantification also enables reliable monitoring of fluctuating bacterial loads (19).

While ddPCR offers superior technical performance, its application as a standalone test for large-scale screening is limited by practical considerations, including significantly higher costs (approximately threefold greater than MeltPro) and longer turnaround times for batch processing. In contrast, MeltPro—a clinically validated and cost-effective method—remains well-suited for rapid initial screening of large sample volumes, despite its reduced sensitivity for detecting low-abundance heteroresistance. To optimize both diagnostic accuracy and resource efficiency, we developed a two-tiered screening strategy: MeltPro serves as an efficient first-line screening tool for population-level testing, while ddPCR is employed for targeted confirmation of equivocal results. This integrated approach leverages the strengths of each method—capitalizing on MeltPro’s affordability and speed for broad screening, while utilizing ddPCR’s enhanced sensitivity to resolve ambiguous samples and reduce false positives—thereby achieving an optimal balance between sensitivity, cost-effectiveness, and workflow efficiency.

We retrospectively collected 73 clinical sputum samples, where MeltPro detected 55 INH-resistant and 18 heteroresistant samples. Interestingly, ddPCR reclassified 7 of the 18 heteroresistant samples identified by MeltPro as INH-sensitive. Combined detection (ddPCR, DST, and Sanger sequencing) confirmed these seven samples were false positives by MeltPro, including one sample identified as *Nocardia* 16S sequencing. These results show ddPCR filters MeltPro false positives, improving INH resistance detection accuracy from 89.04% to 98.63%. This two-tiered strategy thus optimizes the balance between diagnostic accuracy and resource utilization, providing a more practical solution for INH-resistant tuberculosis diagnosis in resource-constrained settings. Positive results from both molecular assays can directly inform the initiation of bedaquiline-containing second-line regimens, reducing treatment delays.

These findings have significant implications for tuberculosis control in China, where MDR-TB burden ranks among the highest globally. Misdiagnoses by traditional methods not only lead to inappropriate use of second-line drugs but also inflate treatment costs to over 100 times that of drug-sensitive patients (27). Our proposed algorithm offers three key benefits: at the individual level, it prevents unnecessary exposure to toxic medications; at the community level, it curbs transmission by promptly isolating true-resistant cases; and at the public health level, it aligns with WHO’s “End Tuberculosis Strategy” by optimizing resource allocation.

However, this study has several limitations. The exclusive inclusion of INH-resistant samples without a sensitive control group precludes the calculation of negative predictive value. The assay’s limited target coverage may miss rare mutations, and the absence of long-term clinical outcome data hinders assessment of treatment efficacy. Future research should expand sample diversity, develop multi-gene ddPCR panels, and conduct prospective cohort studies to evaluate clinical impact.

In summary, our study validates ddPCR’s role as an effective secondary screening tool to improve MeltPro-based tuberculosis diagnostics, especially for heteroresistance detection. While implementation requires careful consideration of cost and throughput, this tiered approach represents a significant step forward in MDR-TB management. Prospective studies integrating next-generation sequencing are needed to fully realize its potential in precision tuberculosis care.

## Data Availability

All data contained in this study can be obtained from the corresponding author upon reasonable request.

## SUPPLEMENTAL MATERIAL

Fig. S1 spectrum.00030-25-s0001.tif ddPCR performance evaluation for different INH resistance mutants.

Fig. S2 spectrum.00030-25-s0002.tif ddPCR LOD validity.

Supplemental legends spectrum.00030-25-s0003.docx Legends for Fig. S1 and S2.

ASM does not own the copyrights to Supplemental Material that may be linked to, or accessed through, an article. The authors have granted ASM a non-exclusive, world-wide license to publish the Supplemental Material files. Please contact the corresponding author directly for reuse.
