# Supplementary figures and images for: Improved detection of isoniazid-heteroresistant Mycobacterium tuberculosis subpopulations by droplet digital PCR compared to MeltPro TB assay
Source: Microbiol Spectr. 2025 Aug 26;13(10):e00030-25. doi: 10.1128/spectrum.00030-25 (PMC12502712; doi:10.1128/spectrum.00030-25)

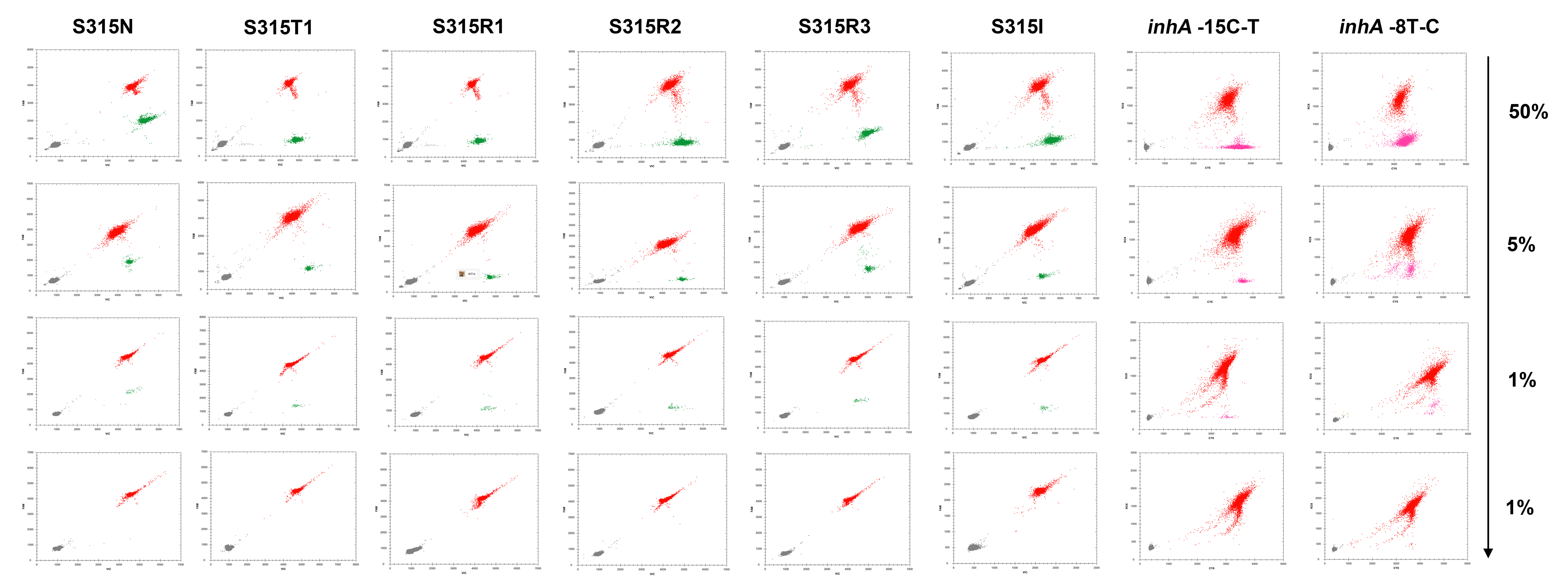

Supplement: Fig. S1 — ddPCR performance evaluation for different INH resistance mutants. [file spectrum.00030-25-s0001.tif]
