# Supplementary material for: Improved detection of isoniazid-heteroresistant Mycobacterium tuberculosis subpopulations by droplet digital PCR compared to MeltPro TB assay
Source: Microbiol Spectr. 2025 Aug 26;13(10):e00030-25. doi: 10.1128/spectrum.00030-25 (PMC12502712; doi:10.1128/spectrum.00030-25)
Supplement: Supplemental legends — Legends for Fig. S1 and S2. [file spectrum.00030-25-s0003.docx]

**Supplement Figure legends**

Supplement Figure 1. DdPCR Performance Evaluation for Different INH-resiatance Mutants

Supplement Figure 2. DdPCR LOD validity
